# Gender differences at presentation of idiopathic pulmonary fibrosis in Sweden

**DOI:** 10.1186/s12890-019-0994-4

**Published:** 2019-11-27

**Authors:** Dimitrios Kalafatis, Jing Gao, Ida Pesonen, Lisa Carlson, C. Magnus Sköld, Giovanni Ferrara

**Affiliations:** 1 Respiratory Medicine Unit, Department of Medicine Solna, Karolinska Institutet, Stockholm, Sweden; 2 Department of Respiratory Medicine and Allergy, Karolinska University Hospital, Stockholm, Sweden

**Keywords:** Idiopathic pulmonary fibrosis, Gender perspective, Interstitial lung disease, Comorbidities, Registry, KBILD

## Abstract

**Background:**

Idiopathic pulmonary fibrosis (IPF) is a disease with poor prognosis mainly affecting males. Differences in clinical presentation between genders may be important both for the diagnostic work-up and for follow-up. In the present study, we therefore explored potential gender differences at presentation in a Swedish cohort of IPF-patients.

**Methods:**

We studied patients included in the Swedish IPF- registry over a three-year period from its launch in 2014. A cross-sectional analysis was performed for data concerning demographics, lung function, 6- min walking test (6MWT) and quality of life (QoL) (King’s Brief Interstitial Lung Disease (K-BILD) score).

**Results:**

Three hundred forty- eight patients (250 (72%) males, 98 (28%) females, median age 72 years in both genders) were included in the registry during the study period. Smoking history (*N* = 169 (68%) vs. *N* = 53 (54%), *p* < 0.05), baseline lung function (Forced vital capacity, % of predicted (FVC%): 68.9% ± 14.4 vs. 73.0% ± 17.7, *p* < 0.05; Total lung capacity, % of predicted (TLC%): 62.2% ± 11.8 vs. 68.6% ± 11.3%, *p* < 0.001) were significantly different at presentation between males and females, respectively. Comorbidities such as coronary artery disease (OR: 3.5–95% CI: 1.6–7.6) and other cardiovascular diseases (including atrial fibrillation and heart failure) (OR: 3.8–95% CI: 1.9–7.8) also showed significant differences between the genders. The K- BILD showed poor quality of life, but no difference was found between genders in total score (54 ± 11 vs. 54 ± 10, *p* = 0.61 in males vs. females, respectively).

**Conclusions:**

This study shows that female patients with IPF have a more preserved lung function than males at inclusion, while males have a significant burden of cardiovascular comorbidities. However, QoL and results on the 6MWT did not differ between the groups. These gender differences may be of importance both at diagnosis and follow- up of patients with IPF.

## Background

Idiopathic pulmonary fibrosis (IPF) is a chronic and progressive disease of unknown etiology limited to the lungs resulting in an irreversible loss of pulmonary function, and, finally, in respiratory failure and death [1, 2]. While results from epidemiological studies have varied due to the different methodologies and diagnostic criteria used, the estimated prevalence of 18–63 cases per 100,000 and a median survival of 3–5 years, makes IPF the most common and deadly form of the idiopathic interstitial pneumonias (IIP) [3–5].

In clinical practice, patients with IPF also present with a large number of comorbidities such as coronary artery disease, arterial hypertension, gastroesophageal reflux, sleep apnea, chronic obstructive pulmonary disease (COPD), asthma and diabetes among others [6], and will often die due to reasons other than IPF [7, 8]. Furthermore, the optimal management of these conditions and their relevance for the prognosis of patients with IPF is still unclear.

In recent years, the gender perspective has become evident in diseases such as COPD [9] or lung cancer [10]. It is well known that IPF is more frequent among males than females [1], but very little is known about potential differences in presentation of the disease and its comorbidities between the genders. Gender was included in a multidimensional index and scoring system for IPF patients, the Gender- Age- Physiology (GAP) score, with males showing a higher risk of dying compared to females [11], but the mechanisms leading to this difference are still poorly characterized. Gender differences may have important implications in the diagnosis, treatment and prognosis of IPF [12] and a better understanding of these differences may also give us new clues about disease etiology.

The main objective of this paper was therefore to explore the gender differences in the clinical features of IPF.

## Methods

### Collection of data

The Swedish IPF Registry was started in 2014. A web-based platform (Granitics Unify Med, Granitics Ltd., Espoo, Finland), allows the registration of demographics, lung function, radiology, health related quality of life (QoL) (assessed with the King’s Brief Interstitial Lung Disease Questionnaire (K-BILD)) [13], ongoing treatments, side- effects and outcomes such as death and lung transplantation at the time of inclusion in the registry and during the follow-up. The platform is currently implemented in 22 respiratory medicine units in the country, including all the main university and reference hospitals. This accounts for approximately 60% of the units with at least one respiratory specialist in service. To be included in the registry, patients had to fulfill the diagnostic criteria for the diagnosis of IPF according to national and international guidelines [1, 14]. Patients are eligible for inclusion in the registry if they have a confirmed diagnosis of IPF by a specialist in respiratory medicine either at an university hospital or a local hospital, and have provided a written informed consent prior to inclusion. No explicit exclusion criteria are defined, but diagnoses other than IPF are not included. Patients were considered incident cases (diagnosis within 6 months from consent) and prevalent if the duration of disease exceeded 6 months.

To explore potential gender differences at the time of inclusion in the registry, a cross-sectional analysis was performed from September 2014 to December 2017 for data concerning demographics, lung function, 6- min walking test (6MWT), and QoL (K-BILD score). Data on comorbidities were mostly self-reported by the patient.

### Statistical analyses

Continuous variables are presented as mean ± standard deviation (M ± SD) or median and range or lower and upper quartile. Categorical variables are presented as proportions of the total population. Two sample t- test was used when appropriate, and non- parametric data was analyzed with the Mann- Whitney test. Results were considered statistically significant if *p* < 0.05. Subgroup analyses for gender were performed. Differences in frequency of comorbidities between genders were expressed with Odds ratios (OR) with 95% confidence interval. Study subjects were pooled to analyze the correlations between quality of life, with K- BILD total score as the dependent factor and clinical variables (age, FVC%, number of comorbidities, BMI, FEV_1_%) in males and females as independent factors. No replacement nor extrapolation of missing values was performed. Univariate analyses included only available variables, while missing values in the multivariable model were automatically removed by the statistical software. Number of available data for each variable is included in respective table. Statistical analyses were performed using the statistical software Wizard Pro (version 1.9.22 (240) @Evan Miller) and SPSS 25.0 (IBM Corporation, Armonk, NY, USA).

The study was approved in August 2014 by the Stockholm’s Regional Ethical Committee (Ref. No. 2014/1202–31/4 and Ref No. 2018/1449–31/1). All patients were included in the registry upon signing a written consent before inclusion.

## Results

### Demographics

Twenty-two respiratory units in the country (61% of the country’s hospitals with a respiratory disease active service) reported data from 348 IPF patients to the Swedish IPF registry during the study period. Demographics at the time of inclusion in the IPF registry are reported in Table 1. Sixty- seven patients (19%) were on anti- fibrotic treatment at inclusion, of whom 14 where incident cases and 53 prevalent.

**Table 1:** Baseline variables of the included patients

Baseline variables	Total
IPF-patients (*N* = 348)	
Incident / Prevalent (N, %)	182 (52.3) / 166 (47.7)
Gender (male /female) (N, %)	250 (71.8) / 98 (28.1)
Age (Median, range)	72.0 (46–88)
BMI (M ± SD)	27.1 ± 4.2
Smoking habit (*N* = 348)	
Never smoker (N, %)	103 (29.6)
Ex-smoker (N, %)	222 (63.8)
Packyear (M ± SD) (Ex-smoker, *n* = 160)	23.0 ± 15.0
Smoker (N, %)	12 (3.5)
Missing info (N, %):	11 (3.2)
Lung function	
FVC% (M ± SD) (*N* = 287)	70.2 ± 15.6
FEV_1_% (M ± SD) (*N* = 307)	76.1 ± 16.6
TLC% (M ± SD) (*N* = 193)	63.9 ± 12.0
DLCO% (M ± SD) (*N* = 221)	46.2 ± 13.9
6-WMT (*N* = 198)	
6-WMD (M ± SD, m)	422 ± 124
B-SpO2% (M ± SD, %)	95.8 ± 2.3
L-SpO2% (M ± SD, %)	86.1 ± 6.7
Comorbidity (*N* = 348)	
Arterial hypertension (N, %)	115 (33.0)
Gastroesophageal reflux (N, %)	113 (32.5)
Other cardiovascular^a^ (N, %)	86 (24.7)
Coronary heart disease (N, %)	67 (19.3)
Diabetes (N, %)	52 (14.9)
Cancer (N, %)	33 (9.5)
Thyroid diseases^b^ (N, %)	22 (6.3)
Sleep apnea (N, %)	22 (6.3)
Asthma (N, %)	15 (4.3)
COPD (N, %)	10 (2.9)
Osteoporosis (N, %)	6 (1.7)
K-BILD (*N* = 292)	53.7 ± 10.7
Anti-fibrotic treatment (N, %)	67 (19.3)
Time from diagnosis to consent (median, Q1- Q3) (months)	4 (Q1:0; Q3: 26)

*N* number(s), *M* mean, *SD* standard deviation, *BMI* body mass index, *FVC%* forced vital capacity, % of expected, *FEV1%* forced expiratory volume in first second, % of expected, *TLC%* total lung capacity, % of expected, *DLCO%* % diffusing capacity for carbon monoxide, *6MWD* 6 min walking distance, *B-SpO2%* Saturation level for oxygen, at rest, *L-SpO2%* Saturation level for oxygen, lowest during/after test, *K-BILD* King’s Brief Interstitial Lung Disease questionnaire

^a^Other cardiovascular disease: Atrial fibrillation and Heart failure

^b^Thyroid diseases: hypo−/hyperthyroidism

Significantly more ex- smokers were reported among males (*n* = 169 (68%); females: *n* = 53 (54%); *p* = 0.020), as well as higher pack years compared to females (24.8 ± 15.1 in male vs. 17.8 ± 13.3 in female, *p* = 0.005) (Table 2). Among ex-smokers, diagnosis of IPF was confirmed more than two decades after smoking cessation in both genders (25 ± 15 years (142 patients) and 24 ± 13 years (46 patients), *p* = 0.796 in males and females, respectively).

**Table 2:** Demographics of included patients divided by gender

	Male	Female
IPF-patients (N, % in total)	250 (71.8)	98 (28.1)
Age (Median, range)	72 (46–88)	72 (56–84)
BMI (M ± SD)	27.0 ± 3.74	27.3 ± 5.18
Smoking status		
Never smoker (N, % in gender)	64 (25.6)	39 (39.8)
Ex-smoker (N, % in gender)	169 (67.6)*	53 (54.1)
Smoker (N, % in gender)	7 (2.8)	5 (5.1)
Missing info (N, % in gender)	10 (4.0)	1 (1.0)
Packyear in ex-smokers (M ± SD)	24.8 ± 15.1*	17.8 ± 13.3

*N* number(s), *M* mean, *SD* standard deviation, *BMI* body mass index

*p < 0.05, male vs. female

### Lung function and six- minute walk test

Forced vital capacity, % of predicted (FVC%) and total lung capacity, % of predicted (TLC%), were significantly lower in males compared to females (Table 3). When the patients were divided by smoking history, male ex-smokers had lower FVC%, TLC% and DLCO%, while no differences were found in never- smokers. Male never- smokers had a longer walking distance (6MWD) than females (*p* = 0.030). No gender- or smoking related difference in other parameters of the 6MWT, including saturation level at rest (B-SpO2) and during/after test (L-SpO2) was observed.

**Table 3:** Lung function parameters, 6MWT and K-BILD results divided by smoking status and gender

	Total		Never smoker		Ex-smoker	
	Male	Female	Male	Female	Male	Female
Lung function						
FVC% (*N* = 287)	*N* = 198	*N* = 89	*N* = 50	*N* = 37	*N* = 139	*N* = 47
	68.9 ± 14.4*	73.0 ± 17.7	69.7 ± 18.0	66.8 ± 16.8	68.7 ± 12.7**	75.8 ± 16.3
FEV1% (*N* = 307)	*N* = 215	*N* = 92	*N* = 54	*N* = 38	*N* = 152	*N* = 49
	76.1 ± 16.1	76.0 ± 17.7	76.8 ± 18.5	71.3 ± 18.9	76.4 ± 14.6	78.6 ± 16.1
TLC%, (*N* = 193)	*N* = 141	*N* = 52	*N* = 35	*N* = 21	*N* = 103	*N* = 31
	62.2 ± 11.8*	68.6 ± 11.3	61.8 ± 11.9	64.4 ± 10.8	62.0 ± 11.7***	71.3 ± 11.0
DLCO% (*N* = 221)	*N* = 158	*N* = 63	*N* = 39	*N* = 25	*N* = 113	*N* = 35
	45.2 ± 13.6	48.8 ± 14.4	49.7 ± 14.1	46.7 ± 14.2	43.8 ± 13.3*	49.3 ± 13.3
6WMT (*N* = 198)	*N* = 139	*N* = 59	*N* = 35	*N* = 22	*N* = 102	*N* = 35
6MWD, (M ± SD, m)	427 ± 128	411 ± 113	439 ± 136*	355 ± 140	426 ± 125	446 ± 79
B-SpO2% (M ± SD, %)	95.7 ± 2.30	95.9 ± 2.3	95.4 ± 2.0	95.8 ± 2.7	95.8 ± 2.4	95.9 ± 2.1
L-SpO2% (M ± SD, %)	85.9 ± 6.44	86.6 ± 7.4	85.8 ± 6.5	88.0 ± 8.5	85.8 ± 6.4	85.7 ± 6.6
K-BILD (*N* = 292)	*N* = 210	*N* = 82	*N* = 55	*N* = 33	*N* = 146	*N* = 44
Psychological	55.1 ± 16.6	54.9 ± 15.6	57.8 ± 16.1	53.0 ± 13.2	54.4 ± 17.0	55.6 ± 17.7
Breathlessness and activities	36.8 ± 18.7	40.0 ± 17.1	38.5 ± 21.9	34.5 ± 13.7	37.2 ± 17.1	41.3 ± 16.7
Chest symptoms	64.1 ± 21.7	66.0 ± 22.9	71.4 ± 21.4*	60.6 ± 20.4	62.5 ± 21.4	67.9 ± 24.2
Total	53.6 ± 10.9	54.3 ± 10.1	55.5 ± 11.1	51.8 ± 7.8	53.3 ± 11.0	55.1 ± 11.3

*M* mean, *SD* standard deviation, *FVC%* forced vital capacity, % of expected, *FEV1%* forced expiratory volume in first second, % of expected, *TLC%* total lung capacity, % of expected, *DLCO%* % diffusing capacity for carbon monoxide, *6MWD* 6 min walking distance, *B-SpO2%* Saturation level for oxygen, at rest, *L-SpO2%* Saturation level for oxygen, lowest during/after test, *K-BILD* King’s Brief Interstitial Lung Disease questionnaire

*: *p* < 0.05, ** *p* < 0.01, *** *p* < 0.001 males vs. females

### Quality of life

The K-BILD total score and its three domain scores for psychological, breathlessness and chest symptoms, were available for 292 (84%, 210 males and 82 females) of the patients. No differences were seen between genders in total K-BILD score, and in the domains of psychological and breathlessness scores (*p* > 0.05) (Table 3). However, the K-BILD domain for chest symptoms were higher in male never- smokers when compared to female never- smokers (*p* = 0.030) (Table 3).

### Comorbidities

Arterial hypertension and gastroesophageal reflux were the two most common comorbidities reported in the whole cohort (Table 1). The frequency of single and multiple comorbidities is presented in Table 4. Multiple comorbidities were observed in 193 males (77% of total males) and 78 females (80%). Coronary heart disease and other cardiovascular diseases (atrial fibrillation and heart failure) were significantly more prevalent among males, while thyroid diseases, asthma and osteoporosis were more prevalent in females. Sub- analyses by gender and smoking history showed that coronary heart disease remained more prevalent in both never- and ex-smoking males compared to females, while gastroesophageal reflux and asthma were more frequent in female never- smokers. Among ex- smoking females, a difference in the prevalence of thyroid diseases was observed. Male ex- smokers showed a higher prevalence of other cardiovascular diseases.

**Table 4:** Frequency of comorbidities reported at inclusion

	Total			Never smoker			Ex-smoker		
	Male	Female		Male	Female		Male	Female	
	(*N* = 250)	(*N* = 98)	OR (95%CI)	(*N* = 64)	(*N* = 39)	OR (95%CI)	(*N* = 169)	(*N* = 53)	OR (95%CI)
Single Comorbidity									
Arterial hypertension (N, %)	84 (34)	31 (32)	1.09 (0.66–1.80)	18 (28)	8 (21)	1.52 (0.59–3.92)	62 (37)	20 (38)	0.96 (0.51–1.81)
Gastroesophageal reflux (N, %)	74 (30)	39 (40)	0.64 (0.39–1.04)	16 (25)	20 (51)	0.32 (0.14–0.74)	55 (33)	17 (32)	1.02 (0.53–1.98)
Other cardiovascular^a^ (N, %)	76 (30)	10 (10)	3.84 (1.89–7.80)	18 (28)	5 (13)	2.70 (0.90–7.88)	54 (32)	5 (9)	4.51 (1.70–12.0)
Coronary heart disease (N, %)	59 (24)	8 (8)	3.48 (1.59–7.58)	14 (22)	1 (3)	10.6 (1.34–84.5)	43 (25)	6 (11)	2.70 (1.07–6.70)
Diabetes (N, %)	38 (15)	14 (14)	1.08 (0.55–2.09)	6 (9)	3 (8)	1.24 (0.29–5.28)	30 (18)	10 (19)	0.93 (0.42–2.05)
Cancer (N, %)	20 (8)	13 (13)	0.57 (0.27–1.19)	4 (6)	5 (13)	0.45 (0.11–1.80)	15 (9)	7 (13)	0.64 (0.25–1.66)
Thyroid diseases^b^ (N, %)	11 (4)	11 (11)	0.36 (0.15–0.87)	4 (6)	2 (5)	1.23 (0.22–7.07)	6 (4)	8 (15)	0.21 (0.07–0.63)
Sleep apnea (N, %)	18 (7)	4 (4)	1.82 (0.60–5.53)	4 (6)	3 (8)	0.80 (0.17–3.78)	13 (8)	1 (2)	4.33 (0.55–33.9)
Asthma (N, %)	5 (2)	10 (10)	0.18 (0.06–0.54)	3 (5)	8 (21)	0.19 (0.05–0.77)	2 (1)	2 (4)	0.31 (0.04–2.22)
COPD (N, %)	8 (3)	2 (2)	1.59 (0.33–7.61)	0 (0)	0 (0)	–	7 (4)	1 (2)	2.25 (0.27–18.7)
Osteoporosis (N, %)	1 (0.4)	5 (5)	0.10 (0.01–0.65)	1 (2)	3 (8)	0.19 (0.02–1.90)	0 (0)	2 (4)	0.06 (0.003–1.30)
Multiple comorbidities									
1- comorbidity (N, %)	77 (31)	31 (32)	0.96 (0.58–1.59)	15 (23)	14 (36)	0.69 (0.28–1.66)	59 (35)	14 (26)	1.50 (0.75–2.97)
2-comorbidties (N, %)	59 (24)	31 (32)	0.67 (0.40–1.12)	13 (20)	13 (33)	0.51 (0.21–1.26)	42 (25)	16 (30)	0.76 (0.39–1.51)
3-comorbidties (N, %)	33 (13)	11 (11)	1.20 (0.58–2.50)	7 (11)	6 (15)	0.68 (0.21–2.18)	26 (15)	4 (8)	2.23 (0.74–6.70)
4-comorbidties (N, %)	20 (8.0)	4 (4.0)	2.04 (0.68–6.14)	5 (8)	0 (0)	7.30 (0.39–136)	14 (8)	4 (8)	1.11 (0.35–3.52)
5-comorbidties (N, %)	4 (2.0)	1 (1.0)	1.60 (0.17–14.3)	1 (2)	0 (0)	1.87 (0.07–47.0)	2 (1)	1 (2)	0.62 (0.06–7.01)

*OR* Odds ratio, *95% CI* 95% confidence interval, *COPD* Chronic obstructive pulmonary disease

^a^Other cardiovascular disease: Atrial fibrillation and Heart failure

^b^Thyroid diseases: hypo−/hyperthyroidism

### Impact factors on K-BILD total score

Multivariate analysis showed FVC% to be an independent predictor for K-BILD total score in males and in total, whereas none of the selected clinical variables were independent predictors for K-BILD total score in females (Table 5).

**Table 5:** Multivariate analysis of K-BILD total score in total group, male and female patients, respectively

	Total, *N* = 240				Males *N* = 165				Females *N* = 74			
	Beta	SE	*P*-value	95%CI	Beta	SE	*P*-value	95%CI	Beta	SE	*P*-value	95%CI
Age	− 0.024	0.096	0.804	−0.213-0.165	− 0.062	0.116	0.595	− 0.291-0.167	0.016	0.182	0.929	−0.347-0.380
BMI	−0.063	0.171	0.714	−0.401-0.275	− 0.307	0.246	0.214	−0.793-0.179	0.178	0.235	0.451	−0.291-0.648
Gender	−0.765	1.53	0.618	−3.779-2.250	–	–	–	–	–	–	–	–
FVC % predicted	0.225	0.084	0.008*	0.060–0.389	0.246	0.107	0.023*	0.034–0.458	0.191	0.138	0.169	−0.083-0.466
FEV1% predicted	−0.03	0.078	0.699	−0.184-0.124	−0.047	0.095	0.618	−0.234-0.140	−0.02	0.143	0.889	−0.305-0.265
Comorbidities status	−0.7	0.546	0.201	−1.775-0.375	−0.865	0.647	0.183	−2.144-0.413	0.06	1.052	0.955	−2.038-2.158

*BMI* Body Mass Index, *FVC%* forced vital capacity, % of expected, *FEV1%* forced expiratory volume in first second, % of expected

*p < 0.05

## Discussion

This paper describes a cohort of IPF- patients included in the Swedish IPF registry in its first 3 years of activity. The study aimed to specifically characterize gender differences in terms of demographics, lung function tests, QoL and comorbidities. The data on demographics, lung function and 6MWT in the whole cohort were similar to what has been reported in other registry studies [15–17]. As in the German INSIGHTS- IPF registry [16], our study showed that IPF occurs approximately 20 years after smoking cessation. This seems to identify a specific risk group which could be a target for screenings for early diagnosis.

The subjects included in this cohort suffered from a mild to moderate IPF. This was reflected by the mean lung function variables presented, such as the mean values of FVC% and diffusing capacity for carbon monoxide, % of predicted (DLCO%). The patients also had a markedly reduced TLC% (Table 3).

Gender is considered an important determinant of outcome in IPF, according to the GAP- index (Gender- Age- Physiology) [11], but it is still poorly characterized which the factors behind this prognostic difference among male and female are. Female patients have a higher FVC% and TLC% than males at inclusion. Notably, the differences were also observed in ex- smokers, where females also presented a higher DLCO%. The higher frequency of ex- smokers among males and the significant difference in pack years might be an explanation to this. No significant differences in lung function were found in never- smokers, further strengthening the notion of cigarette smoking as one of the most important risk factors for the disease and its prognosis.

Furthermore, the study presents the first data on the prevalence of comorbidities among Swedish patients with IPF. Cardiovascular diseases, arterial hypertension, gastroesophageal reflux and diabetes mellitus were the most frequent comorbidities in our cohort. The existence of concurrent diseases aside from IPF, such as cardiovascular disease, could be due to shared predisposing risk factors such as smoking and ageing. These results are comparable to what has been observed in cohorts of other registry- based studies and publications [6, 15–18]. Previous studies have also shown that cardiovascular diseases are more frequent in patients with IPF [18] as well as other ILDs [19] compared to matched controls.

The lower prevalence of COPD and asthma in the cohort compared to other published cohorts [15, 20] may be due to a low use of spirometry in primary care in Sweden [21, 22]. This is likely to reduce the rate of “physician- based” diagnoses of COPD or asthma (without documented spirometry showing airflow limitation) in the general population. Interestingly, females reported a previous diagnosis of asthma 5 times more often than males. It is unclear if this finding reflects a true higher prevalence of asthma in this group, or if early symptoms of IPF were interpreted as “asthma-like” problems, considering the lower number of smokers/ex-smokers among females.

Our study showed significant differences in the prevalence of coronary artery disease, other cardiovascular diseases, thyroid related diseases and osteoporosis between the genders. In addition, almost 80% of patients suffer from one or more comorbid disease. Further studies are needed in order to understand the impact of comorbidities on the course of the disease, e.g. on mortality and survival, QoL and symptoms burden in this cohort. However, studies conducted on the Australian IPF registry (AIPFR) [15] and the German INSIGHTS- IPF [20] have already provided us with some insight into the relevance and influence of comorbidities in IPF. While analyses on survival on the Australian cohort showed no effect on outcome, the cohort in Germany, where almost 90% of patients had one or more comorbidity, showed that the number of comorbidities were associated with mortality. A recent Danish study [19] on comorbidities and mortality in patients with ILDs, showed a significant difference in the frequency of comorbidities and mortality compared to age and gender matched non- ILD controls.

Idiopathic pulmonary fibrosis is known to impair quality of life [23–26]. Our study did not find any significant difference in QoL between genders in terms of K- BILD total score and its domains in the total cohort and in ex- smokers although females presented a more preserved lung function. One reason could be that the questionnaire used in our study (K-BILD) is not sufficiently sensitive to detect differences among males and females. It may also be considered that the way they tend to perceive the disease and their quality-of-life (QoL) is influenced by IPF in different ways. In particular, dyspnea seems to negatively influence physical effort capacity in males, whilst it influences the emotional sphere to a larger extent in females [27]. Interestingly, female never- smokers responded worse on the domain of chest symptoms compared to males. However, the group numbers were relatively small and the result should be interpreted with caution. Perhaps, in females, QoL is impaired earlier during the course of the disease, leading to an earlier diagnosis. This question will need be addressed in future studies.

Furthermore, K- BILD total score was shown to be influenced by FVC%, i.e. a higher value of FVC% was associated with higher total score in males but not in females. In a comparison study [28] of the generic health related quality of life (HRQL) questionnaire EuroQol 5-Dimensional 5- Level (EQ-5D-5 L) and the more ILD- specific K-BILD, regression analysis of impact factors on EQ-5D-5 L and K-BILD results showed similar results on FVC%’ influence on all measures.

Our study has some limitations: First, the data on comorbidities were self- reported by the patients and not instrumentally confirmed, which may result in underreporting. This is a common problem when conducting research on registry- data [15, 20]. Secondly, as data is collected from patients’ routine clinical care from different centers around the country, this might result in variability in the procedures performed. For example, some centers perform a 6MWT and body plethysmography (TLC) more routinely than others.

To date, the information on the impact of treatment of IPF and comorbidities on the course of the disease in males and females, is still lacking, and further research is needed. Moreover, differences in QoL and mortality are poorly explored. Recent studies suggested that optimal management of reflux [29] and sleep apnea may influence the course of IPF [30]. It is likely that instrumental screening of comorbidities soon will become mandatory due to the potential impact of new interventions on survival. Nonetheless, comorbidities in IPF is currently treated as in non- IPF patients, and knowledge about their prevalence is important in order to provide best care and maybe also tailored interventions in consideration of differences on the basis of gender.

## Conclusion

Females have a more preserved lung function than male patients at presentation, while the prevalence of cardiovascular diseases such as coronary heart disease is in increased in males despite smoking history and atrial fibrillation and heart failure in ex- smokers. Meanwhile gastroesophageal reflux and asthma is increased in female never- smokers and thyroid diseases in female ex- smokers. IPF seems to influence quality of life measured with K-BILD differently between the genders, with chest symptoms being more frequent in female never- smokers compared to males. Additional longitudinal research on larger patient cohorts exploring these phenotypic differences are needed in order to elucidate the potential need for tailored therapeutic interventions and overall care on the basis of gender.

## Data Availability

Data supporting the findings of this study are available from the corresponding authors (DK, GF) on reasonable request. The data are not publicly available due to the containment of information that could compromise research participant consent.

## Abbreviations

6MWT: 6- min walking test
BMI: Body mass index
COPD: Chronic obstructive pulmonary disease
DLCO%: Diffusing capacity of carbon monoxide, % of predicted
FEV_1_%: Forced expiratory volume, % of predicted
FVC%: Forced vital capacity, % of predicted
GAP: Gender age physiology
HRQL: Health related quality of life
IIP: Interstitial idiopathic pneumonia
ILD: Interstitial lung disease
IPF: Idiopathic pulmonary fibrosis
K- BILD: King’s Brief Interstitial Lung Disease
QoL: Quality of life
TLC%: Total lung capacity, % of predicted
UIP: Usual interstitial pneumonia
